# Adipic acid: A green and sustainable crosslinker for innovative food packaging applications

**DOI:** 10.1016/j.fochx.2025.103476

**Published:** 2026-01-08

**Authors:** Reza Abedi-Firoozjah, Tina Alighadri, Swarup Roy, Arezou Khezerlou, Mahmood Alizadeh Sani

**Affiliations:** a Student Research Committee, Department of Food Science and Technology, School of Nutrition Sciences and Food Technology, Kermanshah University of Medical Sciences, Kermanshah, Iran; b Student Research Committee, Department of Food Science and Technology, Faculty of Nutrition and Food Sciences, Tabriz University of Medical Sciences, Tabriz, Iran; c Department of Food Technology and Nutrition, School of Agriculture, Lovely Professional University, Phagwara 144411, Punjab, India; d Nutrition Research Center, Tabriz University of Medical Sciences, Tabriz, Iran; e Department of Food Science and Technology, School of Nutritional Sciences and Dietetics, Tehran University of Medical Sciences, Tehran, Iran; f Nutraceutics Research Center, Tehran University of Medical Sciences, Tehran, Iran

**Keywords:** Green packaging, Crosslinkers, Adipic acid, Mechanical properties, Barrier characteristics

## Abstract

Biopolymer-based food packaging films are a promising eco-friendly alternative to conventional petroleum-based plastics. Adipic acid (AdA) has gained attention as a green and effective crosslinker for improving the performance and characteristics of biopolymer films. AdA (a dicarboxylic acid) is abundantly found in sources such as beets and sugarcane. Its nontoxic nature makes it a suitable candidate for food packaging. Despite its potential, no comprehensive review has studied the progress of applying AdA as a crosslinker in food-oriented biopolymer packaging. To address this gap, this review provides a comprehensive analysis of chemistry and recent advances in the integration of AdA with various polymers for food preservation. This review specifically details the developments in AdA-increased polysaccharide/protein/biopolyester-based packaging films and their role in food preservation. The addition of AdA to biopolymer films improved their mechanical strength, barrier properties, and thermal stability owing to covalent and hydrogen bonds, while also introducing antioxidant activity that helps extend the shelf life of packaged foods. In summary, AdA-crosslinked biopolymer films represent a viable and eco-friendly pathway for food packaging innovation, addressing environmental concerns and consumer demand for safe, sustainable materials.

## Highlights

• Adipic acid (AdA) emerges as a sustainable crosslinker, offering an ecofriendly alternative for packaging films.
• AdA improves mechanical, thermal, and barrier properties through interaction with biopolymers.
• The formation of hydrophobic ester bonds in films, extending the shelf life of food products.
• The crosslinking efficiency depends on AdA concentration, reaction temperature, and pH.

## Graphical abstract

**Figure:**
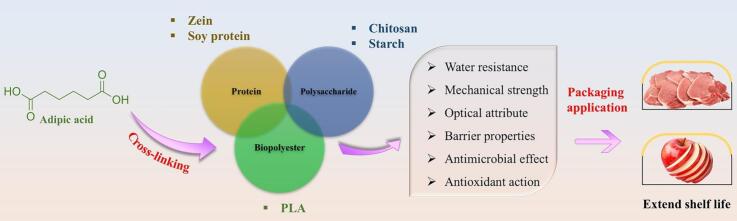

## 1 Introduction

Conventional plastic packaging poses severe environmental and health risks due to toxic emissions during incineration and persistent microplastic pollution (Meng et al., 2023). Bio-based polymers have emerged as sustainable alternatives; however, their adoption is limited (<1 % market share) due to their poor mechanical strength, weak water resistance, and inferior barrier properties (Abedi-Firoozjah et al., 2024; Deng et al., 2022). To overcome these challenges, several strategies have been developed to enhance the properties of biodegradable films, including the preparation of composite or multilayer food packaging films (FPFs) (Akti, 2025), incorporation of nanomaterials (Ahmad et al., 2025) and bioactive compounds (Li et al., 2025), polymer blending (Qader et al., 2025), microencapsulation of functional ingredients (Bansal et al., 2025), biopolymer modification (Norozi et al., 2025), and the use of crosslinking agents (Sani et al., 2024; Wen et al., 2021). Crosslinking with bio-derived agents is one of the most effective strategies for overcoming these drawbacks.

Biopolymer crosslinking can be achieved through physical (e.g., temperature, radiation), chemical (e.g., green and synthetic), or biological (e.g., enzymatic) methods (Khezerlou et al., 2025; Sani et al., 2024). This process involves the formation of covalent or ionic bonds between polymer chains, significantly enhancing the structural integrity, thermal stability, and barrier properties (e.g., against water vapor and oxygen) of bio-based packaging materials, thereby improving their performance in food packaging applications (Wang et al., 2025).

Adipic acid (AdA), a non-toxic and low-hygroscopic compound, has gained increasing attention as a green crosslinker for FPFs (Sole et al., 2022). Although traditionally produced petrochemically, AdA can now be obtained sustainably from agricultural waste (starch, hemicellulose, lignin), fully aligning with green chemistry principles and the transition towards a bio-based economy (Rios et al., 2021). AdA, extensively utilized in producing polymers, polyurethanes, nylon 6,6, plasticizers, pharmaceuticals, and lubricants (Lang & Li, 2022), also functions in the food industry as a food additive, an acidulant for pH regulation, and a flavor enhancer to improve taste (Sorathiya et al., 2025). Additionally, it aids in gel formation and viscosity control in confections and gelatin desserts, enhancing their texture and stability (Zakeri et al., 2023).

Compared to other green crosslinkers, AdA offers distinct advantages due to its linear, aliphatic six‑carbon backbone, which imparts enhanced flexibility and superior mechanical properties to the resulting polymer networks (Falamarzpour et al., 2017; Sun et al., 2018). Its non-toxic nature and role in forming covalent ester bonds further contribute to improved water resistance and reduced water vapor permeability in various systems. AdA primary constraint is the relatively short, linear dicarboxylic structure, which provides fewer functional groups per molecule compared to multifunctional crosslinkers like citric acid (tri-carboxylic) or tannic acid (poly-phenolic) (Sani et al., 2025). This can result in a lower crosslinking density and a less rigid three-dimensional network in systems requiring high structural integrity (Zhang, Jiang, et al., 2022). Furthermore, while it forms stable covalent esters, physical or ionic crosslinks involving AdA in some matrices may demonstrate instability upon prolonged exposure to aqueous environments, potentially limiting its utility in highly hydric applications (Abdulhameed, Abdullah, et al., 2025). Therefore, while AdA is a highly effective green crosslinker for enhancing mechanical flexibility and hydrophobic character, its performance is system-dependent, and it may be less suitable than more highly functional alternatives for applications demanding extremely dense or hydrolysis-resistant networks (Sole et al., 2022, Abdulhameed, Al Omari, et al., 2025, Abdulhameed, Khan, et al., 2025).

This study reviews the use of AdA as a crosslinker in FPFs. It reviews the chemistry, structure, and crosslinking mechanisms of AdA in protein/polysaccharide/biopolyester-based film matrices. This review analyzes how AdA enhances key film properties, such as mechanical strength, thermal stability, and barrier performance, and explores its functional role in active packaging. Finally, the practical applications of AdA-modified films in food preservation are evaluated, and future challenges and prospects in this field are discussed.

## 2 AdA; chemistry, structure, properties

**Fig. 1:**
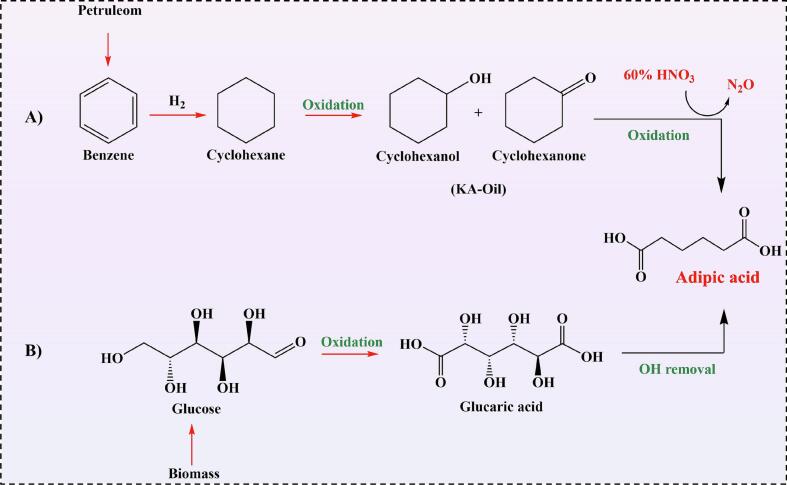
Conventional petroleum-based (A) and green/sustainable (B) pathways for the production of AdA. (For interpretation of the references to color in this figure legend, the reader is referred to the web version of this article.)

AdA, known as hexanedioic acid, is a dicarboxylic organic compound with the chemical formula C_6_H_10_O_4_ (Fig. 1). With a molecular mass of 146.14 g/mol and pKa values of 4.43 and 5.41, AdA is one of the most significant linear-chain aliphatic acids. It is soluble in ethanol, acetone, and water (up to 24 g/L), with solubility increasing as the temperature increases. In its anhydrous form, AdA molecules form pairs linked by hydrogen bonds between the -COOH groups of each monomer (Sorathiya et al., 2025).

AdA is usually produced either through petroleum-based or green method extracted from agricultural waste/biomass and renewable biomaterials including: 5-hydroxymethylfurfural; glucose; γ-valerolactone; phenolic compounds; mucic acid and succinic acid, by a variety of ways: chemical, biological and biochemical processes (Rios et al., 2021). Renewable resources help to reduce the overall petroleum dependency and environmental concern. Chemical approaches typically involve catalytic oxidation of precursors, while biological and biochemical routes utilize microorganisms and enzymes to convert biomass-derived molecules into AdA under milder, eco-friendly conditions. This variety of synthesis pathways highlights the versatility and potential of bio-based platform molecules for producing environmentally sustainable AdA (Cronjé et al., 2023; Lang & Li, 2022; Zhang et al., 2025).

In the chemical production of AdA, it is produced from a mixture of two oils, cyclohexanol and cyclohexanone, which are referred to as ketone-alcohol oil (KA-oil). After the ketone-alcohol oil is oxidized in several steps, AdA is produced. The side products of this reaction are nitrogen oxide, glutaric acid, and succinic acid. Nitrogen oxide is a greenhouse gas that leads to the destruction of the ozone layer. Therefore, the chemical production of AdA is not a suitable method and is harmful to the environment (Rios et al., 2021).

Extracting AdA from agricultural waste or biomass, especially lignin, the main source of AdA is considered an environmentally friendly and sustainable approach. This process utilizes by-products generated during agricultural production, such as crop residues and lignocellulosic materials, which would otherwise be discarded or burned, causing environmental pollution. By converting these wastes into valuable chemicals like AdA, not only is waste minimized, but the need for fossil-based resources is also reduced, making the overall process greener and more sustainable (Lang & Li, 2022; Liu et al., 2024). Additionally, AdA is produced in nearly all aerobic organisms through the tricarboxylic acid cycle (Zhou et al., 2020, Lang & Li, 2022). AdA, recognized by the International Energy Agency (IEA) as the most significant dicarboxylic acid, is a promising platform chemical for bio-based production. Each year, approximately 3 million tons of AdA are produced worldwide, with a market value approaching $6 billion and a compound annual growth rate of 3–5 %. Most AdA is used in the nylon industry, with smaller amounts employed in the production of plasticizers and polyurethanes (Lang & Li, 2022; Rios et al., 2021). Additionally, it has applications in various other sectors, such as the food industry (as a food additive and crosslinker) and pharmaceuticals (Lang & Li, 2022). Recent innovations focus on improving selectivity and reducing environmental impact using advanced catalysts such as metal-organic frameworks (MOFs) and ionic liquids. For example, a catalyst based on phosphotungstic acid encapsulated in UiO-66 MOF achieved an 80.3 % yield under mild conditions, with high stability over multiple cycles (Feng et al., 2019). These modern chemical methods aim to minimize energy consumption and harmful byproducts and offer sustainable, environmentally friendly alternatives to traditional petrochemical routes for AdA production.

The biochemical production of AdA involves microbial fermentation of renewable biomass, offering an environmentally friendly alternative to chemical methods. Genetically engineered microbes, such as *Escherichia coli* (Zhou et al., 2020) and *Saccharomyces cerevisiae* (Zhang, Cheng, et al., 2020), are used to convert sugars like glucose and xylose into AdA (Wu et al., 2023). For example, *E. coli* has been reprogrammed to produce 4.97 g/L AdA after 72 h of fed-batch fermentation processes that eliminate the need for chemical inducers, making the process more sustainable (Yuan et al., 2025). Similarly, *S. cerevisiae* has been engineered to produce AdA at a titer of approximately 2.59 mg/L, converting sugars into intermediates like muconic acid, which can be hydrogenated into AdA (Raj et al., 2018). Enzymatic catalysis also offers a sustainable route, where specific enzymes convert bio-derived substrates such as fatty acids into AdA under mild conditions (Ju et al., 2020). These biochemical methods highlight the potential for scalable, low-impact AdA production from renewable resources.

Overall, biotechnological routes present a viable and ecofriendly alternative for AdA production, with continuous progress making industrial-scale application increasingly feasible.

**Fig. 2:**
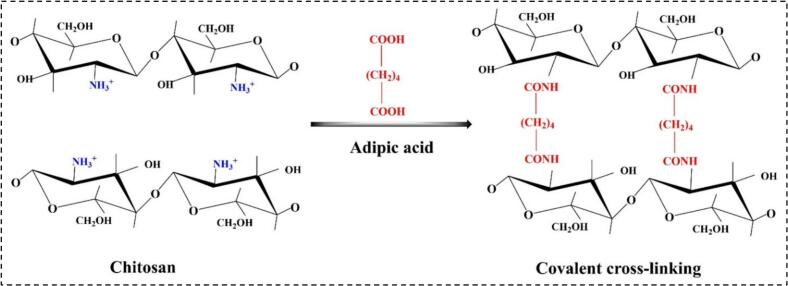
The molecular interaction between AdA and chitosan.

AdA is highly valued as a crosslinker in the packaging industry because its carboxyl group can react with various biopolymers (Sole et al., 2022). As a renewable resource, AdA supports ecofriendly packaging by enhancing the mechanical strength of biopolymers and improving compatibility with different materials (Sam, 2020). However, challenges such as high production costs, processing complexities, and potential environmental impacts require careful management (Sole et al., 2022). Crosslinking restricts biopolymer mobility, resulting in a compact, dense structure that reduces gas permeability, increases tensile strength (TS), crystallinity, and thermal stability, while decreasing elasticity (Sam, 2020; Sole et al., 2022; Zhang, Shen, et al., 2022). Additionally, heat application enhances the ability of AdA to form crosslinks. For instance, in chitosan-based films, the amino groups of chitosan interact with the carboxyl groups of AdA at high temperatures, as shown in Fig. 2 (Zhang, Jiang, et al., 2022).

**Fig. 3:**
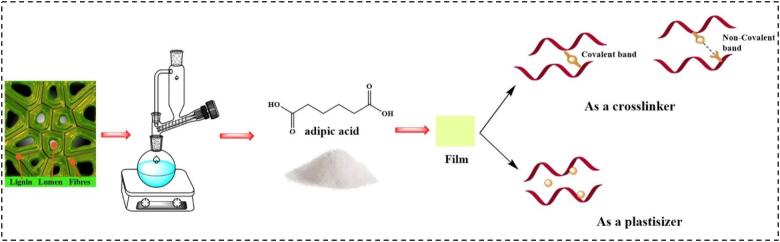
AdA derived from lignin and application in packaging film as crosslinker and plasticizer.

In composite films, biopolymers interact through AdA (as a crosslinker and plasticizer) (Fig. 3), which not only enhances the physical and mechanical properties of the films but also improves their chemical and functional characteristics (antioxidant and antimicrobial activities) (see in Table 1) (Zhang, Shen, et al., 2022). AdA can improve the antioxidant properties by donating hydrogen ions to neutralize free radicals (Vibha & Negi, 2020). Additionally, the accumulation of AdA hydrogen ions near microbial cell membranes alters their permeability, enhancing the antimicrobial effects (Vibha & Negi, 2020; Zhang, Jiang, et al., 2022). This makes AdA highly valuable and widely used in the packaging industry. Beyond packaging, AdA serves as an acidifier, flavoring agent, and gelling component in the food industry (Mahmoudi Kharazm & Safaei-Ghomi, 2024).

**Table 1:** The effect of AdA on the physico-mechanical, structural, and functional properties of biopolymer-based FPFs.

Film matrix	AdA content	Functional properties after adding AdA	Structural properties	Barrier properties	Mechanical strength	Ref.
SPI	NM	NM	Morphology; Improve surface smoothness, strengthened spatial network, and preservation of a compact, intact internal structure	WVP; 32.46 → 24.98 g·mm/m^2^·d·kPa))	EB; 50 → 15 % TS; 5 → 3.5 MPa	(Xu et al., 2018)
CS	0.5 % and 1.0 % *w*/*v*	NM	Morphology; smoother, less porous Crystallinity; more ordered structure Higher thermal stability, elevated decomposition temp, strong covalent bonds	WVP; 1.27 → 0.12 **×** 10^−7^ g m^−1^ h^−1^ Pa^−1^	NM	(Sole et al., 2022)
CS/zein	3 % (w: w)	Antimicrobial activity	Morphology; Improve nanofiber uniformity, compatibility, and structural integrity FTIR; Peak at around 1715 cm^−1^ (esterification) and hydroxyl shift to 3375 cm^−1^ (hydrogen bonding) Thermal stability; Decrease mass loss to around 85 %	NM	EB; 28.36 → 42.26 % TS; 5.99 → 9.54 MPa	(Zhu et al., 2019)
Corn starch	0–25	NM	Better compatibility among compounds	NM	EB; 2.2 → 18.1 (after 60 days) TS; 24.3 → 26.4 MPa	(Zhang, Cheng, et al., 2020)
Gliadin-EC	5 % w/v	DPPH; 85.4 % Against *S. aureus* and *E. coli* up to 80 % lose un cell viability	Morphology; Improved spinnability, fiber uniformity, conductivity and solution viscosity. FTIR; Peaks at 3450 cm^−1^ and 2937 cm^−1^ indicating hydrogen bonding Thermal stability; Improved via crosslinking and interactions among biopolymers	NM	NM	(Hosseini et al., 2021)
Starch	1 % wt	NM	Thermal stability; covalent amide bonds and more durable films	NM	EB; 4.9 → 5.84 % YM; 653.17 → 533.79 MPa TS; 7.58 → 8.73 MPa	(Mahmud, 2022)
SPI	1 % and 1.5 % *v*/v		Morphology; Display rougher fracture surfaces and enhanced stretchability. Improved adhesion and crosslink network formation.	NM	TS; 4 → 6 MPa) YM; 55 → 120 MPa EB; 24 → 26 %	(Sellivam et al., 2016)
PVA/CNC	5 % wt	NM	FTIR; Indicate hydroxyl, silanol, and amine group vibrations, with characteristic peaks	NM	YM; 30.4 → 49.4 MPa TS; 20.6 → 37.6 MPa	(Nuradibah et al., 2018)
CS	0.73 g	DPPH; 52.19 %	Morphology; smoother, fewer holes Crystallinity; increased and more ordered structure Better thermal stability and longer-lasting films	WVP; 22 → 13.07 g mm/m^2^ d kPa	EB; ∼4 → ∼8 % TS; 85 → 48 MPa	(Zhang, Jiang, et al., 2022)
CS	NM	Against *S. aureus*, *E. faecium,* and *S. agalactiae* up to 28 mm inhibition zone	FTIR; Induces amino group ionization, carboxylate formation, and crosslinking without amide bond formation.	WVP; 1.50 → 0.23 g mm/m^2^ d kPa)	TS; 13.80 → 16.65 MPa EB; 5.17 → 8.43 %	(Medimagh et al., 2016)

AdA; adipic acid, CNC; cellulose nanocrystals, NM; not mentioned, TS; tensile strength, EB; elongation at break, WVP; water vapor permeability, YM; Young's modulus, CS; Chitosan, SPI; soy protein isolate, EC; ethyl cellulose, CNC; cellulose nanocrystal, PVA; polyvinyl alcohol,

The following sections explore the chemical nature of AdA and related considerations, as well as discuss in detail how adding AdA to packaging films made from carbohydrates, proteins, and other biopolymers affects their properties and the underlying mechanisms of action.

## 3 Mechanism of AdA in packaging films as a crosslinker

The use of AdA as a biodegradable and natural crosslinker is an innovative approach to enhance the physical, mechanical, and thermal properties of bio-based films (Sam, 2020; Sole et al., 2022). AdA forms a robust three-dimensional polymer network by establishing covalent or non-covalent bonds between polymer chains, either within the same molecule (intramolecular) or between different molecules (intermolecular) (Hammer et al., 2021). The effectiveness of these crosslinks depends on several key factors, including the structure and molecular weight of the biopolymers, the presence of reactive functional groups, and the compatibility of AdA with the biopolymer matrix. These elements collectively determine the strength and nature of the interactions (Gautam et al., 2021).

**Fig. 4:**
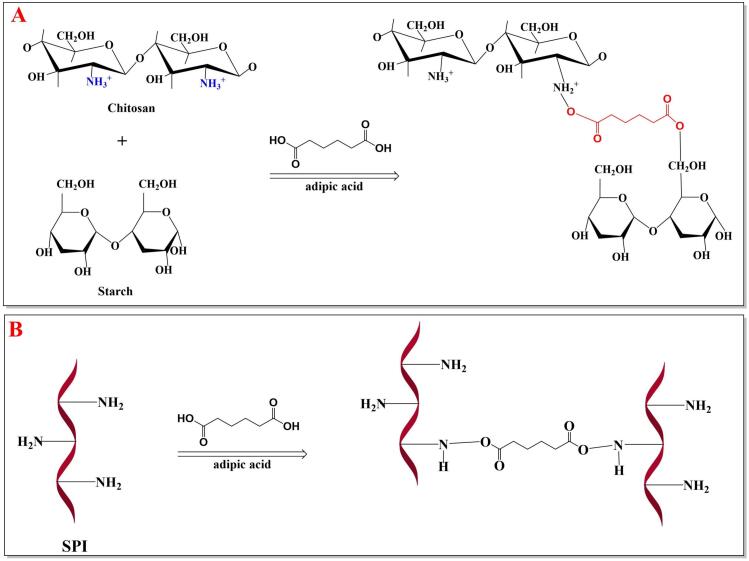
Crosslinking process between AdA and starch/chitosan (A) and soy protein isolate (SPI) (B).

The crosslinking process enhances the structural integrity of the bio-based films by reducing chain mobility, resulting in a denser and more compact matrix (Khezerlou et al., 2025; Sani et al., 2025). This leads to improved tensile strength, reduced gas and water vapor permeability, and greater thermal stability, making the films ideal for sustainable packaging applications (Sani et al., 2025; Shaddel et al., 2025). This improvement occurs because proteins, polysaccharides, and bio-polyesters contain active functional groups, such as amino (−NH_2_), carboxyl (-COOH), hydroxyl (-OH), and carbonyl (C=O), which participate in various reactions (Sani et al., 2025). For instance, AdA's carboxyl groups can react with hydroxyl or amino groups in biopolymers like proteins, starch, nanocellulose or chitosan, forming strong chemical bonds such as ester or amide linkages (Fig. 4) (Zhang et al. 2022, Sani et al., 2025). These interactions not only strengthen the film but also enhance its resistance to environmental factors like gas, moisture and heat (Khezerlou et al., 2025; Sole et al., 2022).

Moreover, the role of AdA as a crosslinker extends beyond mechanical improvements. By facilitating a uniform network, it can enhance the incorporation of active agents, such as antioxidants or antimicrobials, into the film matrix, further improving the film's functionality (Vibha & Negi, 2020, Zhang et al. 2022). However, achieving optimal crosslinking requires careful consideration of processing conditions, such as temperature and pH, as well as AdA concentration, to ensure compatibility and avoid undesirable side reactions (Sole et al., 2022). Therefore, AdA's ability to form tailored interactions with biopolymers makes it a versatile and eco-friendly option for advancing the performance of bio-based packaging films.

In conclusion, AdA enhances the mechanical and structural properties of protein- and polysaccharide-based films through multiple reactions. It forms ester bonds between its -COOH groups and the -OH/NH_2_ groups of proteins and polysaccharides, reducing surface tension and creating a condensed film structure. This condensation, along with the replacement of hydrophilic groups with hydrophobic ester groups, forms a tortuous pathway that lowers water and gas permeability while increasing tensile strength (TS) and flexibility, and reducing the contact angle on the film surface (Sole et al., 2022; Vibha & Negi, 2020). Additionally, AdA promotes crosslinking via nucleophilic addition of chitosan or protein -NH_2_ groups to carbonyl groups and disulfide bond formation between sulfhydryl groups in proteins, which increases the film's resistance to high temperatures and pH levels. Together, these interactions create a stable and durable network, significantly improving the physical and mechanical properties of bio-based food packaging materials.

In a study, Xu et al., 2018 found that AdA, used as a crosslinker, effectively improved the properties of soy protein isolated (SPI) and kapok husk films (Xu et al., 2018). The addition of a glycerol-based biological polyester (BGP) with AdA as a plasticizer notably enhanced the tensile strength of these films, rendering them more durable. They emphasized that incorporating BGP significantly enhanced the mechanical performance of SPI films by promoting better crosslinking and stability. The TS of the BGP-incorporated films initially increased for the first 20 days owing to crosslinking and Maillard reactions, but then decreased significantly (*p* *<* *0.05*) over time, with TS decreasing by 9.28 %, 4.00 %, and 14.29 % in SPI-BGP I, II, and III films after 60 days, respectively, compared to a 22.22 % decrease in the glycerol-only films. BGP contributed to better TS retention, with SPI-BGP films showing higher stability than pure glycerol films. The addition of AdA as a cross-linker further affected TS; moderate AA levels improved tensile strength, whereas excessive AdA reduced interactions among protein chains, decreasing TS. The EAB also declined over 60 days, with control films losing 72.83 %, whereas SPI-BGP films showed less reduction: 49.2 %, 35.56 %, and 52.5 % for BGP I, II, and III, respectively. BGP enhanced EAB stability by forming hydrogen bonds with SPI, creating a network structure that increased its elasticity.

Accordingly, a more profound analysis of how reaction conditions (pH, temperature, catalysts, etc.,), influence the reaction pathways (esterification vs. amidation) and the final network architecture is needed in furture studies.

## 4 Biopolymer based FPFs crosslinked with AdA

### 4.1 Protein-based FPFs

One of the types of biodegradable packaging is protein-based packaging (Abedi-Firoozjah et al., 2024). Plant proteins derived from sources such as amaranth, chia seeds, quinoa, mushrooms, and potatoes offer promising options for developing sustainable packaging materials (Senthilkumaran et al., 2022). These proteins exhibit diverse interactions due to their complex secondary, tertiary, and quaternary structures, which enhance their functionality in film production. Furthermore, protein-based films demonstrate appropriate water vapor and oxygen barrier properties, while also exhibiting desirable mechanical characteristics. This makes them an effective choice for food packaging applications, contributing to both sustainability and performance (Thivya et al., 2024). Soy protein and zein are the most widely used proteins used in protein-based FPFs crosslinked with AdA.

#### 4.1.1 Soy protein

Soy proteins are a mixture of albumins and globulins, most (90 %) of which are storage proteins with spherical structures. The presence of polar amino acids, such as arginine, lysine, and cysteine,  in the soy protein structure leads to their interaction through carboxyl, -OH, and amine groups (Zheng et al., 2022). For example, AdA was used in a soy protein-based film with a kapoki shell. Increasing the AdA content from 0 % to 1.0 % increased the TS and Young's modulus (YM) of the soy protein-based biocomposite layers from 4 to 6 MPa, while the elongation at break (EB) decreased from 24 to 20 %. However, the AdA concentration of 1.5 % decreased the TS and YM of the film but increased the elongation at break. EAB decreases with increasing AdA, but slightly recovers at 1.5 % because AdA acts as a plasticizer, enhancing flexibility and weakens the matrix. This is probably because all the functional groups of the matrix and filler have fully interacted with the AdA, and the excess AdA is placed between the chains of the film, causing a lubrication effect in the film (Sellivam et al., 2016).

#### 4.1.2 Zein

Corn protein is a biodegradable material that dissolves in organic solvents due to its hydrophobic amino acids, such as phenylalanine, leucine, alanine, and proline. Its -NH_2_ and -COOH groups allow it to form chemical bonds. The unique ability of proteins to create a flexible network has made them a popular choice for biopolymer-based packaging materials (Bayer, 2021). However, a key challenge with protein-based films is their high moisture sensitivity (Gupta et al., 2022). To address this, researchers often use crosslinking agents like AdA to improve the mechanical strength and reduce the WVP of these films. AdA is particularly favored because of its own biodegradable properties (Gautam et al., 2022). Accordingly, the effect of AdA in the zein-based film with chitosan was investigated (Cheng, Wang and Weng, 2015). The film containing AdA showed a significant reduction in permeability, decreasing from 0.71 g.mm/cm^2^ in the control film to 0.22 g.mm/cm^2^ in the crosslinked film. Indeed, AdA serves as a water vapor barrier binder in film matrix. Further, modified films showed improved mechanical resistance, increasing from 1.353 MPa to 1.873 MPa. After adding AdA to the film, the antioxidant properties of the film increased from 4.08 to 98.61 %, and the antimicrobial activity of the film containing AdA against *S. aureus* and *E. coli* was higher than that of the control film. Therefore, the use of AdA is a promising technique to improve the performance and application of protein-based packaging materials, especially considering their sensitivity to water, which hinders many of their potential applications (Cheng et al. 2015).

### 4.2 Polysaccharide-based FPFs

Significant progress has been achieved in creating biodegradable products derived from polysaccharides, like cellulose, chitosan, starch, pectin, alginate, carrageenan, pullulan, and gums. These biopolymers are promising materials in the quest for more sustainable and biodegradable packaging solutions, offering functionality while contributing to environmental conservation (Zhu, 2021). Among these, chitosan and starch are the most commonly used natural biopolymers in the fabrication of biodegradable crosslinked FPFs.

#### 4.2.1 Chitosan

Chitosan, a biopolymer derived from the deacetylation of chitin, is the second most abundant natural polysaccharide after cellulose and the most prevalent animal-origin biopolymer (Abedi-Firoozjah et al., 2025). Chitosan's functional groups (NH/NH₂, OH, C=O, C-O-C) enable its use in film production, mainly through hydrogen and ionic bonding via the C-O-C group (Abedi-Firoozjah et al., 2025). However, pure chitosan-based films often exhibit stiffness and limited mechanical strength, restricting their industrial applications.

To overcome these limitations, crosslinking chitosan with AdA via amidation reactions enhances film properties (Abdulhameed et al. 2025). The -NH₂ groups of chitosan react with the -COOH groups of AdA, forming amide bonds (-CONH-) that improve flexibility, stiffness, and TS (Oladzadabbasabadi et al., 2024). In another study, incorporating 0.05 % AdA into chitosan/zein films significantly improved physical and mechanical properties, with WVP decreasing from 0.71 to 0.022 g.mm/cm^2^/day, TS increasing from 1.353 ± 0.226 MPa to 1.608 ± 0.147 MPa, and EB rising from 3.185 ± 2.083 % to 22.31 ± 7.231 % (*P < 0.05*). Notably, AdA did not significantly affect film thickness, indicating strong compatibility with biopolymers and improved thermal stability (Cheng et al., 2015). Similarly, AdA in chitosan/nanocellulose films increased TS from 60 to 113 MPa due to hydrogen bonding and amidation between AdA's -COOH and chitosan's -NH₂ groups (Falamarzpour et al., 2017).

In another study, chitosan-based films were produced using various bio-based dicarboxylic acid solutions, including succinic acid, adipic acid, suberic acid, and sebacic acid. The influence of these non-toxic acids on the water vapor barrier, mechanical, and antimicrobial properties of the chitosan films was investigated. It was found that films made with acetic acid had significantly higher water vapor permeability (WVP) and lower tensile strength (TS) and elongation at break (%EB) compared to those prepared with dicarboxylic acids (*p* < 0.05). When adipic acid or suberic acid solutions were used, the resulting chitosan films showed an 85 % reduction in WVP, a 21–27 % increase in TS, and more than a 60 % improvement in %EB than the acetic acid-based films. Additionally, chitosan films modified with either adipic acid or suberic acid demonstrated antimicrobial activity against all tested microorganisms (Medimagh et al., 2016).

#### 4.2.2 Starch

Starch is a biodegradable polymer consisting of amylose and amylopectin (Cheng et al., 2021). With the help of moisture and heat, starch is converted into a thermoplastic material for film production (Matheus et al., 2023). Starch-based films are sensitive to moisture due to their -OH groups (Frangopoulos et al., 2023); however, they can form films and coatings with good inhibitory properties against the transfer of gases like oxygen and carbon dioxide (Thakur et al., 2019). The combination of different biopolymers can strengthen the physical and mechanical properties of the starch film (Cheng et al., 2025), but in order to better interact with biopolymers, AdA is used as a binder in films (Sam, 2020; Wongphan et al., 2025). Indeed, one of the most prevalent modifications of starch is its crosslinking, aimed at enhancing its functionality for diverse uses (Cheng et al., 2025). AdA as a key crosslinker, interacts with at least two hydroxyl (-OH) groups within starch molecules, forming bonds that result in both crosslinked starches (Silva et al., 2013, Sun et al., 2018). The use of AdA in carbohydrate-based films can partially solve the physical and mechanical problems of the film (Spiridon et al., 2011, Cruz et al., 2015). For example, The films incorporating adipic acid demonstrated the greatest light transmittance (36.27 % at 600 nm) and the lowest permeability to water vapor (1.16 × 10^−10^ g.m.m^−2^.s^−1^.Pa^−1^) and oxygen (3.81× 10^−16^ cm^3^.cm.cm^−2^.s^−1^.Pa^−1^). Those cross-linked with boric acid achieved the highest tensile strength, whereas films cross-linked with citric acid showed the greatest elongation at break. Thermogravimetric analysis (TGA) indicated that the cross-linked films possessed improved thermal stability compared to the control films. Fourier transform infrared (FTIR) analysis verified that the cross-linking agents were involved in intermolecular interactions, such as grafting or cross-linking, between starch and polyhydroxyalkanoate (PHA) (Sun et al., 2018).

AdA for chitosan-starch composite films, enhancing their stability under environmental stresses (Wan Yusof et al., 2024). The incorporation of AdA increases mechanical and water resistance and reduces WVP in chitosan-starch films (Yang et al., 2024). AdA typically increases TS by strengthening intermolecular interactions (Mahmud, 2022). For example, Mahmud et al. reported that AdA crosslinking in arrowroot starch films increased TS from 7.83 to 8.58 MPa and reduced moisture absorption from 150 % to 60 %. This improvement stems from ester bonds reducing hydrophilicity by limiting free -OH groups, creating a denser polymer network (Mahmud, 2022).

Therefore, according to the studies conducted, AdA can be used as a binder in carbohydrate-based films. On the other hand, another application of AdA is its softening properties. In fact, carboxylic acids have been known as softeners for years (Cruz et al. 2015). Because they are non-toxic and safe, they are also used as softeners in the packaging industry (Vikhareva et al., 2022). In the European Union and the United States, they are an alternative to softeners such as dibenzoate and phthalates (Zhang et al., 2023).

### 4.3 Biopolyester-based films crosslinked by AdA

Biodegradable plastic packaging, including polyethylene (PE), bio-based polyethylene terephthalate (PET), polybutylene adipate-*co*-terephthalate (PBAT), and polyhydroxyalkanoates (PHA), is increasingly approved for its ability to decompose under specific conditions such as optimal humidity, pH, temperature, and oxygen availability, thus reducing environmental impact (Arman Alim et al., 2022; Cheng et al., 2024; Din et al., 2020). The blending of these biopolymers with natural biopolymers, such as starch, has been shown to enhance the biodegradability of packaging films. However, films made solely from natural biopolymers often exhibit inappropriate physical and mechanical properties. A key challenge in combining starch with PBAT is the lack of interaction between starch and PBAT can weaken composite film structure, but using AdA as a crosslinker forms ester bonds, enhancing mechanical and barrier properties for better packaging applications (Silva et al., 2013, Menzel, 2020). For example, the aim of Silva et al. study was to investigate the compatibilizing effect of AdA (at concentrations of 0.5 %, 1.0 %, and 1.5 % *w*/w) on starch/glycerol/poly(butylene adipate-*co*-terephthalate) (PBAT) films produced via reactive extrusion. The addition of AdA as a crosslinker led to improvement in tensile strength (increasing from 7.28 to 8.45–9.13 MPa), elongation at break (notably with 0.5 % adipic acid, from 516.51 % to 679.10 %), and Young's modulus (from 21.72 to 28.75 MPa), as well as a decrease in water vapor permeability (from 9.76 to 8.32 × 10^−11^ g/s.m.Pa) compared to the control film without the AdA. Additionally, films containing AdA showed a microstructure with fewer cracks. However, the thermal degradation and crystallinity profiles of the films were not affected by the addition of the AdA (Silva et al. 2013).

The mechanical properties and tear resistance of an environmentally friendly flexible packaging film made from poly(lactic acid) (PLA) and poly(butylene adipate-*co*-terephthalate) (PBAT) were examined using the blown film extrusion method. The use of PLA and PBAT in packaging is often restricted due to their inherent brittleness, low stiffness, and poor compatibility. Accordingly, the influence of different plasticizers including adipate, adipic acid, glycerol ester, and adipic acid ester on the plasticization of PLA and the production of PLA/PBAT blown films was thoroughly assessed. The findings revealed that a plasticizer with both ether and ester groups (specifically, adipic acid ester) significantly enhanced the flexibility of PLA and its compatibility with PBAT. The incorporation of this plasticizer increased the chain mobility within the PLA matrix and improved the interfacial adhesion between the plasticized PLA and PBAT. As a result, the plasticized PLA/PBAT blown films produced by blown film extrusion showed notable improvements in tear resistance, rising from 4.63 to 8.67 N/mm in the machine direction and from 13.19 to 16.16 N/mm in the transverse direction (Kim et al., 2020).

## 5 Applications of AdA crosslinked FPFs

The development of advanced biodegradable packaging to extend the shelf life of perishable foods is an important objective in food technology. A promising strategy involves using AdA as a crosslinking agent in biopolymer-based films. This approach significantly enhances the film's barrier and functional properties, making it highly effective for food preservation (Gebresas et al., 2023). For example, zein/chitosan films crosslinked with AdA and fortified with phenolic acids (e.g., ferulic and gallic acid) exhibit strong antibacterial activity against both *S. aureus* and *E. coli* (Cheng et al., 2015).

The antimicrobial mechanism is attributed to the action of COOH groups from AdA, which disrupt bacterial cell membranes, leading to leakage of intracellular components and cell death (Hauser et al., 2016). Notably, inhibitory effects were more pronounced against *S. aureus* than *E. coli*, with inhibition zones increasing by approximately 50 % and 30 %, respectively, upon AdA incorporation. This enhancement is attributed to a synergistic interaction between AdA and phenolic compounds, which exceeds the inherent antimicrobial properties of chitosan films. Thus, AdA serves not only as a crosslinking agent improving film integrity but also as an antimicrobial enhancer, making it a valuable component in active food packaging applications (Cheng et al. 2015).

Research on AdA as a crosslinker in biopolymeric FPFs is still limited, despite its promising potential. Currently, only a few studies have explored its applications in this field. One study focused on using AdA to develop biopackaging for pork meat, enhancing food safety. Zhu and *co*-workers engineered polyethylene oxide (PEO)/*Dendrobium officinale* polysaccharide (DOP) nanofibers at different ratios (PEO: DOP, 3:1, 3:2, 3:3), with AdA added as a crosslinker to enhance fiber properties (Zhu et al., 2019). Nanofibers with DOP ratios of 3:1 and 3:2 showed limited antibacterial effects, but at higher DOP ratios of 3:3 and a3:3, bacterial counts significantly decreased (*p* *<* *0.05*) after 48 h, with a3:3 achieving 6.68 CFU/g compared to 7.11 CFU/g without AdA. The addition of AdA enhanced DOP release, reaching 70.29 % after 12 h, about 14 % higher than nanofibers without AdA, due to improved fiber structure. High DOP content caused structural defects, reducing porosity and release, but AdA maintained a well-structured fiber matrix, boosting antibacterial agent release. Sensory evaluation showed that pork treated with nanofibers (3:3 and a3:3) and stored at 25 °C for 2 days, retained better color, texture, elasticity, odor, and viscosity, and was preferred by consumers. The inclusion of AdA significantly improved both antibacterial activity and release behavior, making these films promising for extending meat shelf life.

**Fig. 5:**
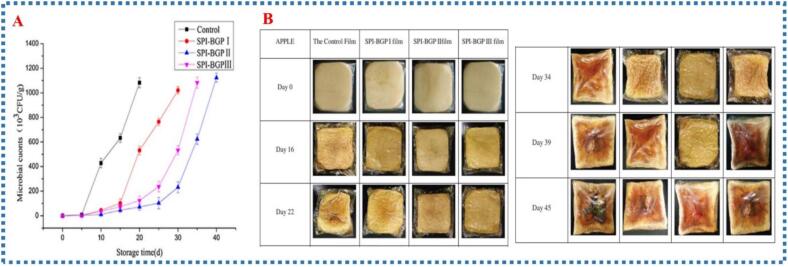
Microbial counts during storage in the four types of films **(A).** Visual changes of fresh cut apples when wrapped with SPI-based films **(B).** Reprinted from (Xu et al. 2018) with permission from Wiley Online Library.

Another investigation explored its role in preserving the freshness and quality of apple slices over time. Xu et al. proposed SPI films plasticized with bio-poly glycerol (BGP) at ratios 1:2, 1:1, and 2:1, with AdA incorporated to enhance properties for fresh-cut apple packaging (Fig. 5) (Xu et al. 2018). When applied to fresh-cut apples, these films extended shelf life by reducing oxygen transfer, which helped prevent browning due to SPI-BGP's oxygen barrier. Microbial analysis showed that microbial counts increased over time, reaching 1.08 × 10^6^ CFU/g after 20 days in samples wrapped with glycerol-plasticized film, indicating quality loss. In contrast, apples wrapped with SPI-BGP films exhibited significantly lower microbial counts (*p* *<* *0.05*), maintaining better quality. The shelf life was extended to 28, 34, and 40 days for apples wrapped in SPI-BGP I, II, and III films, respectively. This indicates that SPI-BGP films effectively control gas and moisture transfer, limiting bacterial growth by blocking contact between the apple surface and air. Overall, the films preserved the freshness and quality of the apples much longer than standard packaging, highlighting their potential in fresh produce preservation. The addition of BGP notably enhanced the films' barrier and antimicrobial properties, contributing to prolonged shelf life and better quality retention.

Given that the findings of these studies provide useful information, it is not sufficient evidence to support definitive statements regarding the efficacy of AdA as a means to an end for food preservation, or the potential for AdA as a method of food preservation on a larger scale. The current trend towards greater demand for sustainable methods of food preservation indicates that additional thorough investigation will need to take place across different types of foods and storage locations to substantiate the claims associated with AdA as a new material of food preservation.

## 6 Safety aspects of AdA

The safety of AdA is complex and depends on factors such as the route of exposure, the dose, and the solvent use. Oral exposure studies show low toxicity. For example, in a group (unspecified number) of humans who received a daily oral dose of 100 mg AdA/kg for eight days, or in rats, rabbits, or hamsters when administered orally, no abnormal signs and developmental toxicity were observed (Fiume et al., 2020). But AdA dust irritates eyes, skin, mucous membranes of lungs and nose. Threshold of human eye irritation with AdA is 20 mg/m^3^ (Nehring et al., 2020).

There are no reports of mortality following dermal administration of AdA, and rabbits tolerated doses estimated at 100–250 mg/kg in studies designed primarily to investigate irritation. Further, a single acute inhalation exposure to 31 mg/L AdA for 1 h or 7.7 mg/L for 4 h, no lethality occurred. Acute use of AdA is slightly to moderately toxic to fish, daphnia and algae (Fiume et al., 2020). In toxicity studies, rats fed diets containing up to 5 % AdA (equivalent to 2.7, 280, 809, and 1302 mg/kg-bw/day) showed minimal effects, with slight reductions in food intake and body weight at the highest doses (Luttrell & Klaassen, 2016). Inhalation exposure at 5.41–7.61 mg/m^3^ for 4 h resulted in no mortalities but caused respiratory irritation (Kennedy Jr, 2002). Dermal exposure in rabbits led to redness and pain, but no deaths. Long-term carcinogenicity studies over two years indicated no carcinogenic effects. Reproductive toxicity assessments in rats and mice revealed no adverse effects even at high doses (up to 263 mg/kg in pregnant mice) (Luttrell & Klaassen, 2016).

There were also no signs of toxicity in young rats given up to 3400 mg/kg or adult rats up to 2400 mg/kg for four weeks, or daily oral doses of 100 or 200 mg/kg (in ethanol) after five weeks (Vikhareva et al., 2021). As mentioned, the toxicity of AdA depends on various factors like the solubility of AdA. According to studies, when the solvent used was acidic or non-polar (oil), it led to better solubility of AdA, as a result, it participated in the body's metabolism and was converted into 2-ethylhexanoic acid. If AdA remains insoluble, it will not participate in metabolism and will be excreted through urine.

Overall, AdA demonstrates low toxicity with high safety margins, and no significant carcinogenic or reproductive risks have been identified. The current safety assessment focuses mainly on short-term toxicity, and to date, there has not been enough data to estimate the safety of longer-term exposure, the potential for migration from food contact materials, or the ability of food contact materials to comply with regulations. These factors are critical to a complete risk assessment and comply with regulations (Kennedy Jr, 2002). AdA is stable at ambient condition, however, when combined with oxidizers and/or metals, it will react violently with an exothermic reaction creating flammable gases, which are captured in non-metallic containers.

Scientific investigation showing there is no possible transfer of AdA from food packaging to general distribution. Migration remains well below the level determined to be safe for humans, confirming AdA's continued ability to be safely applied to FPFs (Ubeda et al., 2020).

Migration studies on multilayer packaging confirmed that levels of AdA and related compounds stay below regulatory thresholds (Úbeda et al., 2017). To safeguard consumer health, EFSA also established a specific migration limit (SML) for AdA, which is designed to prevent its migration into food from exceeding levels deemed safe for human consumption. This limit generally restricts the amount of AdA that can transfer into food to a maximum of 5 mg/kg of food. Adherence to these regulatory migration thresholds is obligatory for all food contact materials used within the European Union, ensuring that their use remains within safe and approved parameters (EFSA Panel on Food Contact Materials and Aids 2015).

## 7 Limitations and future perspectives

The primary limitations of AdA in (FPFs) stem from safety concerns related to migration and potential food contamination. Precise control of its concentration is crucial to ensure safety while preserving the film's performance and integrity, including mechanical properties, permeability, thermal stability, and overall functionality.

Environmental factors like light and heat can degrade AdA and reduce its biological activity; encapsulation enhances stability, bioavailability, and enables controlled release, prolonging its effectiveness.

Early results show strong potential for AdA-based FPFs, but research is limited on water resistance (e.g., contact angle and moisture absorption), release/diffusion characteristics, sensory evaluation, and consumer acceptance. These films remain in the research phase, requiring collaboration and investment for commercial viability, including scaled manufacturing adaptations and migration management.

Methods like solvent casting and electrospinning are unsuitable for large-scale production; future efforts should focus on extrusion and reactive blending, along with improved surface modifications for uniform nanofiller dispersion. Upcoming developments may include smart features, optimized formulations for strength/flexibility/barrier properties/biodegradability, hybrid composites with synergistic nanofillers, and solutions to regulatory/scaling challenges.

AdA's limited thermomechanical properties can be improved via branching agents, chain extenders, and process optimization. While AdA biodegrades naturally, modifications with fillers or compatibilizers can alter this property. Thus, standardized biodegradation testing is crucial before claiming environmental breakdown capabilities after modifications.

## 8 Conclusion

AdA emerges as a suitable, green, and safe material for fabricating biodegradable FPFs significantly enhancing their overall performance. The imperative to transition towards sustainable materials underscores the industrial potential of AdA-crosslinked FPFs as a viable alternative to conventional plastics. The influence of AdA on film properties is primarily concentration-dependent, enabling precise tuning of the film's structure. At optimal concentrations, it forms a continuous and dense crosslinked network by compressing and condensing the film matrix. This structure restricts particle movement, thereby markedly improving the film's physical and mechanical properties. Crosslinking can be physical (induced by heat) or chemical, involving hydrogen, ionic, and covalent bonds.

Beyond structural enhancement, AdA incorporation improves the biological functionality of films, imparting antioxidant and antimicrobial activities. Its acidic nature is particularly beneficial, as the carboxyl group can solubilize acid-soluble polymers, thereby improving miscibility among diverse biopolymer components. To conclude, these improvements in physico-chemical properties directly contribute to extending the shelf life of packaged foods.

## CRediT authorship contribution statement

**Reza Abedi-Firoozjah:** Writing – original draft, Methodology, Conceptualization. **Tina Alighadri:** Writing – original draft, Methodology, Investigation, Conceptualization. **Swarup Roy:** Writing – review & editing, Investigation. **Arezou Khezerlou:** Writing – original draft, Methodology, Investigation, Conceptualization. **Mahmood Alizadeh Sani:** Writing – review & editing, Methodology, Conceptualization.

## Declaration of competing interest

The authors declare that they have no known competing financial interests or personal relationships that could have appeared to influence the work reported in this paper.

## Data Availability

The data that has been used is confidential.
